# The CRIT framework for identifying cross patterns in systems biology and application to chemogenomics

**DOI:** 10.1186/gb-2011-12-3-r32

**Published:** 2011-03-31

**Authors:** Tara A Gianoulis, Ashish Agarwal, Michael Snyder, Mark B Gerstein

**Affiliations:** 1 Department of Genetics, 77 Ave. of Louis Pasteur, Harvard Medical School, Boston, MA 02115, USA; 2 Wyss Institute for Biologically - Inspired Engineering, 3 Blackfan Circle, Boston, MA 02115, USA; 3 Department of Computer Science, Yale University, 51 Prospect St, New Haven, CT 06511, USA; 4 Department of Molecular Biophysics and Biochemistry, Yale University, 266 Whitney Ave, New Haven, CT 06511, USA; 5 Department of Genetics, Stanford University School of Medicine, Alway M344, Stanford, CA 94305, USA; 6 Program in Computational Biology and Bioinformatics, Yale University, 266 Whitney Ave, New Haven, CT 06511, USA

## Abstract

Biological data is often tabular but finding statistically valid connections between entities in a sequence of tables can be problematic - for example, connecting particular entities in a drug property table to gene properties in a second table, using a third table associating genes with drugs. Here we present an approach (CRIT) to find connections such as these and show how it can be applied in a variety of genomic contexts including chemogenomics data.

## Background

Understanding the relationship between two or more variables is a driving motivation of many biological questions. The past several decades has seen a rapid increase in our ability to discern such relationships at multiple levels from molecular to cellular to whole populations. However, our ability to understand the relationships between different scales and different types of data is still limited [1].

**Figure 1:**
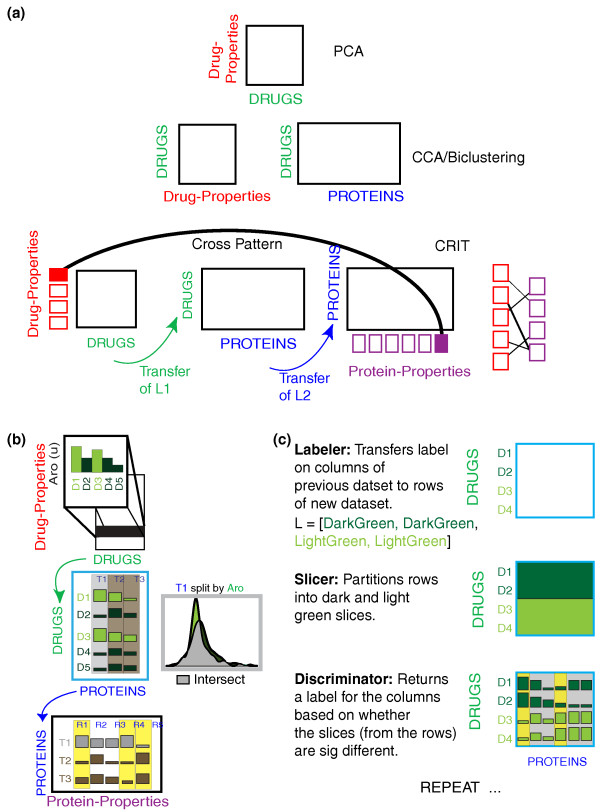
**Difference between CRIT and previous techniques**. **(a) **Data in a single matrix can be investigated using techniques such as PCA. Techniques such as CCA are applicable to two matrices with a common index. CRIT allows working with three or more matrices that do not share a common index. **(b) **An overview of CRIT. **(c) **A simple example showing how proteins can be labeled as sensitive to a particular drug property. See text for more details.

Here we introduce Cross Pattern Identification Technique (CRIT) as a means of integrating at least three matrices which do not all share the same index. The goal of CRIT is to systematically combine information from multiple tables with different indices allowing one to not only stack features in a single dimension but also to span across multiple ones. Thus, CRIT captures a new type of relationship between different types of data (for example drugs and their protein targets) which we term a 'cross pattern.' What is a cross pattern and how does this differ from the more traditional integration methods? There are two main differences: (1) It preserves the underlying structure of the individual datasets allowing for greater transparency and more importantly (2) it does not rely on a single index for querying. In other words, cross patterns are conceptually related to correlation but are not correlations as there is no obvious way to correlate two differently indexed objects. To better illustrate these differences, in Figure 1, we are given three pieces of information: the properties of a set of drugs, the properties of a set of proteins, and which drugs targeted which proteins. Our goal is to determine if there are any properties of drugs that are related to any property of the protein target. As a test query, in Figure 1b, we narrow our question to *Which types of proteins are disrupted by aromatic drugs? *Understanding these types of relationships could provide additional details about general mechanisms of drug-protein binding and how to design drugs to disrupt a particular function. Investigating this question though would require integration across two different object types: proteins and drugs.

As shown in Figure 1a, principal component analysis (PCA) captures the set of drug properties with the most variance, but without further collapsing of the tables, it is not possible to discern what types of proteins are most affected by aromatic drugs. Similarly, both canonical correlation analysis (CCA) and biclustering can define relationships amongst datasets that share the same index [2,3]. Namely, they can identify relationships between either drug properties and their protein targets or protein properties and their drug targets but cannot span across a differently indexed dataset. Although methods are available for integrating more than three matrices when all share the same index variable (see discussion in [4]), how to integrate features when they do not all share the same index remains an open question. We suggest that cross patterns provide the flexibility and intuitiveness to allow for the formal definition of these types of relationships. In the remainder of the text, we describe CRIT and apply it to three different types of problems: breast cancer gene expression, yeast regulatory networks, and a further explication of the above example in chemogenomics data. Example datasets, code, and documentation for CRIT can be found at [5].

### Algorithm

#### Cross-integration (CRIT)

Figure 1b shows an overview of the entire method and Figure 1c illustrates the individual functions of CRIT. CRIT has three generic types of functions: a labeler, a slicer, and a discriminator. The labeler transfers a label from one dataset to another (rows to columns or the reverse). The slicer partitions this new dataset into separate 'slices' on the basis of the label generated in the previous step. Finally, the discriminator applies a statistical test to the slices to generate a new set of labels. More generally, the discriminator determines if there are any features in the second dataset that 'discriminate' among the labeled slices based on the parameter in the first dataset. The entire process is iterated until all of the matrices have been used.

In the instance in Figure 1b, c, the first label is generated by simply assigning each drug to be aromatic or not aromatic. Next, this label is transferred via the labeler to the second matrix containing the drugs and their associated protein targets. The slicer partitions this matrix into two slices (aromatic and non-aromatic drug treatments). Finally, the discriminator examines if the label is meaningful for any of the protein targets. If aromaticity were significant in determining the disruptiveness of a particular drug to that protein, one should see two distinct fitness populations as shown in Figure 1b. However, should this label be non-discriminatory that is the aromaticity of the drug is not a factor in determining its effectiveness on the protein of interest, the label should not split the drug treatments into distinct populations. Those proteins which illustrated sensitivity to the aromaticity of the drug are then labeled aro-sensitive and this label is propagated to the next matrix and so on.

## Results and Discussion

### Overview

Below, we applied CRIT to three different types of problems: extracting general trends from properties of transcription factors and their associated targets in the yeast regulatory network, relationships between gene properties such as expression and binding status and breast cancer type, and finally using chemogenomics, chemoinformatics, and functional genomics data we investigated the relationship between properties of drugs and properties of their associated targets. In all cases, we differentiate between three different levels of significance in discussing the individual cross patterns. The level of confidence in each cross pattern is further distinguished by the thickness of the line as shown in each of the three result figures (see Additional file 1 for investigation of method robustness using synthetic datasets).

### Regulation: transcription factors and their target properties

Cis-regulatory elements as a means of regulating gene expression have been extensively studied. However, beyond such motifs, are there inherent properties of the targets themselves that make them more or less likely to be regulated by a given class of transcription factors (TFs)? As an example, do essential transcription factors preferentially regulate essential targets? Are there genome composition features such as GC or codon bias that influence which targets are regulated by which TFs?

There is no meaningful way of correlating properties of TFs on top of properties of their downstream targets as the number of targets of each TF is variable. These two objects do not share the same index. However, despite the dissimilarity of object types, such integration is critical to identify principles governing transcriptional regulatory evolution as such patterns would not be observable from just looking at a single TF or single set of targets.

#### Datasets

Nineteen transcription factor and gene target properties were taken from an extensive meta-analysis in [6] (Additional file 2). A genome-wide mapping of transcription factor and targets as defined in [7] was used as the connector matrix. The intersection between TFs mapped by Harbison *et al. *and TF and protein properties from Xia *et al. *resulted in 201 TFs and 5,125 gene targets.

#### Evaluating significance

For each TF property, TFs were labeled as either above or below median value (given the number of TFs, breakdown into finer classes yielded numbers too small to perform meaningful statistics). This label was then transferred to the connector matrix where the rows represented the individual transcription factors and the columns potential gene targets. Each element of this matrix was a score of how likely the TF would be to regulate the specific target. The rows of this matrix were then partitioned via the labeling generating two different distributions of gene target scores. The likelihood that the scores were obtained from the same distribution was evaluated using Welch's t-test and *q *values were generated through FDR-correction of associated *P *values. Those targets with *q *< 0.05 were considered to be more likely to be regulated by one type of TF than another are defined as TF-property (for example essentiality-sensitive) targets. This label (sensitive/insensitive) was applied to the columns of the TF/target matrix and propagated to the rows of the target/target-property matrix. The process was then repeated where the target/target-property matrix was partitioned on the basis of sensitivity and those target properties that were able to discriminate between the TF property-sensitive targets and TF property-insensitive targets. The end result was a set of cross patterns connecting a specific property of a transcription factor to a specific property of a target.

#### Results

**Figure 2:**
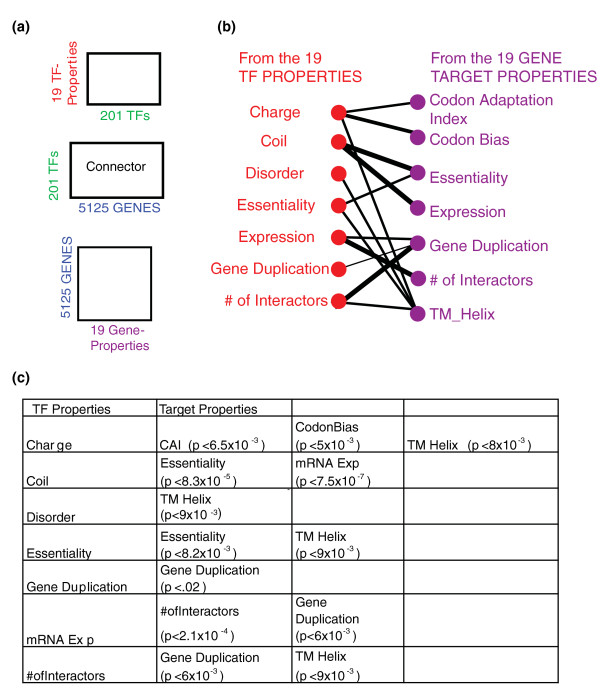
**Regulatory network cross patterns**. **(a) **Three matrices integrated in the regulatory network example. **(b) **Lines connecting properties of a TF and its associated targets represent the cross patterns identified. Three line thicknesses correspond to differing levels of significance of the cross pattern: thickest *P *< 10^-4^, thicker *P *< 10^-3^, and thin *P *< .05. **(c) **Summary table including the significance scores for each cross pattern reported.

In total, we identified 13 significant cross patterns relating properties of TFs and properties of targets suggesting an overall pattern of these TFs exhibiting 'preferences' or 'sensitivities' to particular attributes of targets (Figure 2).

Many of these cross patterns were between the physicochemical and composition properties of TFs and targets suggesting that the composition and evolutionary history of the gene target may be a useful complement to the presence or absence of a given motif in predicting transcription factor binding.

As an example, we identified a subset of seven transcription factors that exhibited a strong preference for either essential or inessential targets (*q *< 0.05, FDR-corrected). One-hundred-thirty-five targets were preferentially regulated by either an essential or nonessential TF. The number of protein-protein interaction partners of a given TF was connected to the level of gene duplication of the genes the TF targeted. In addition, TF expression was also connected to the level of gene duplication.

### Breast cancer: ER status and ER binding

In our second application, we applied CRIT to a well characterized system. Estrogen receptor (ER) activation is one of the primary molecular features used to differentiate breast cancer subtypes through immunohistochemical staining. Activation of this receptor results in strikingly different cancer phenotype due to extensive downstream remodeling of transcriptional programs, and the genes and molecular mechanisms affected by this dichotomy are of particular interest. Identification of gene signatures of specific tumor types is critical in the development of more targeted therapeutics. van't Veer and colleagues identified two breast cancer subtypes distinguished by differences in the immunohistochemical stain for estrogen receptor (ER). Further, through supervised methods they identified 550 additional genes that were signatures of this status [8].

#### Datasets

Maps of ER to target genes were obtained from [9]. Definition of target defined as in [9]. ER status, microarray data, and patient metadata were all taken from [8].

#### Evaluating significance

A slight modification of CRIT was required to accommodate binary features. We used the hypergeometric distribution in order to calculate the significance of overlap of differentially expressed ER+ and ER- genes. To be explicit, the problem can be described in terms of determining the probability of drawing *x *white balls from an urn of *m *white balls and *n *black balls after taking out *k *balls. Thus, we regard the ER binding genes as the total number of white balls(*x*) and non-binding genes as black balls (*n*). The total number of differentially expressed genes (ER+ vs ER-) represents the sample withdrawn and *x *of these are also ER targets (that is sampled white balls). Thus, we calculate the significance of overlap by summing *P*(*X *>= *x*).

#### Results

**Figure 3:**
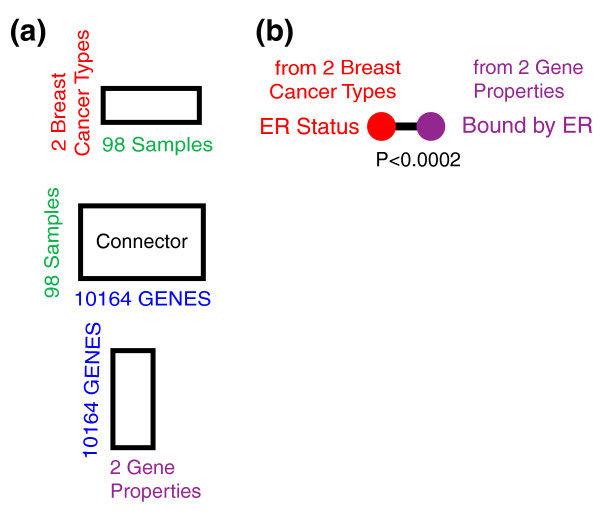
**Breast cancer cross patterns**. **(a) **Three matrices integrated in the breast cancer application. **(b) **A single cross pattern was identified.

We applied CRIT to the van't Veer patient metadata, signature genes, and estrogen binding information from Carroll *et al. *[9] (Figure 3a). In this manner, we were able to recapitulate the observed relationship between ER(+) tumors and the expression of genes that are bound by estrogen (*P *< 2 × 10^-4^) (Figure 3b). Although this application serves as an important validation, the result is already well known. To show the potential of CRIT, we applied it to a more complex problem domain.

### Chemogenomics: drug properties and target properties

**Figure 4:**
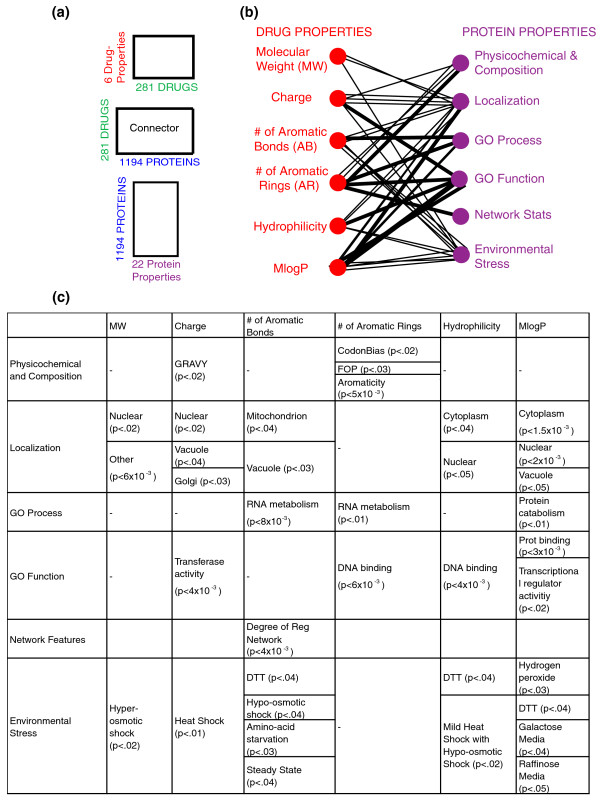
**Chemogenomics cross patterns**. Analogous to Figure 3. **(a) **Three matrices integrated in the chemogenomics network example. **(b) **Lines connecting properties of a drug and properties of its associated targets represent the cross patterns identified. Three line thicknesses correspond to differing levels of significance of cross pattern: thickest *P *< 10^-3^, thicker *P *< 0.01, and thin *P *< 0.05. **(c) **Summary table including the significance scores for each cross pattern reported.

To investigate more complex non-obvious connections, we applied CRIT to identify relationships between small molecule properties and properties of their protein targets (Figure 4a). Numerous papers have attempted to find relationships between particular drugs and particular targets [10-12]. Here, we investigated a slightly different question. Rather than looking at individual drugs and individual targets, we examined whether there are classes of drugs that are particularly disruptive to a class of proteins.

As an example, we tested the hypothesis that the subset of proteins bound or more indirectly affected by a structural parameter may also share physicochemical or other types of properties by posing questions in the form: *Do positively charged proteins exhibit a tendency to interact with negatively charged compounds?*

#### Datasets

Hillenmeyer *et al. *tested 291 unique compounds on the heterozygous yeast deletion collection under a number of different concentrations (Additional file 1). We selected profiles generated using the minimum drug concentration since specificity decreases as drug concentrations approach toxicity. Small molecules were converted to text strings called SMILES [13] (Additional file 3) and small molecule properties were computed [14] (Additional file 4, 5). Only compounds with no missing values were kept, resulting in 281 unique compounds.

Yeast strains with defects in transport machinery, lipid permeability, and drug efflux pumps, and so on [15] were removed from the connector matrix as in [16] as such mutants are affected by drugs in a non-specific manner [17]. Analogously, if the variance of a single target's growth scores across all small molecule perturbations is too low, one would only be in the noise. Only ORFs which had a variance of growth scores across the different drug treatment greater than 1.5 were included. After removal of ORFs missing values in the target-feature datasets (see below), 1,170 ORFs remained. Finally, there were a few cases where the ORF grew better in the presence of the drug, suggesting resistance. In this analysis, we do not investigate this scenario.

Physicochemical properties were obtained from SGD including molecular weight, isoelectric point, protein length, GRAVY (hydropathicity index), and aromaticity [18] as were the gene composition features (codon adaptation index (CAI) and frequency of optimal codons (FOP)) and GO categories [19]. The localization data was taken from [20]. We used two types of networks: protein-protein interactions and gene regulatory [21] (genetic interaction and phosphorylome [22] had too few nodes to determine significance). All topological statistics (degree, clustering coefficient, betweenness, eccentricity, shortest path) were computed for each node in the network using tYNA [23]. The environmental stress response data were taken from [24].

#### Evaluating significance

For each drug property, drugs were labeled as either above or below median value. This label was then transferred to the connector matrix where the rows represented the individual drugs and the columns represented a protein. Each element of this matrix was a fitness defect score measuring the level of disruptiveness of a particular drug treatment on a particular protein target.

For each protein, we considered whether the protein's disruption (as measured by fitness defect) is significantly different when subjected to the lo- versus hi-labeled drugs by computing a sensitivity score:

where the numerator is the difference of the mean growth scores for a protein treated with drugs labeled as high and low, and the denominator is simply the difference between the standard error for high and low. Welch's t-statistic was used to compute *P *values, and proteins with *P *< 0.05 were considered sensitive to the particular drug property (DP) used for the partitioning (see Additional file 1).

For each continuous-valued protein property, we computed a sensitivity score as shown above. Localization is a categorical variable requiring special treatment to generate the sensitivity score. This variable was first transformed to a series of binary features where each compartment was treated as a separate feature (one if the protein was localized to the compartment of interest and zero otherwise). Enrichment for a particular localization category was determined via the hypergeometric distribution.

#### Results

**Figure 5:**
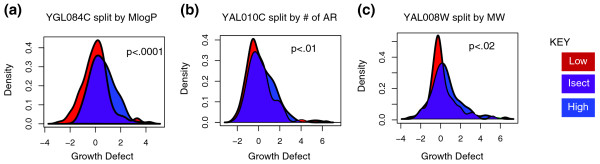
**Plots of DP-sensitive proteins**. The x-axis is the growth defect score of the particular protein after treatment with a small molecule and the y-axis is the density plot. The purple region shows the overlap between the two distributions. The smaller this overlap the more 'sensitive' the protein is to the value of the particular drug property. **(a) **YGL084C or GUP1 is involved in glycerol uptake. Treatment with drugs with a low partition coefficient have a significantly larger fitness defect (*P *< 0.0001). **(b) **YAL010C (MDM10) is involved in importing and assembling beta barrel proteins. It is significantly more disrupted by drugs with fewer aromatic bonds (*P *< 0.01). **(c) **YAL008W or FUN14 is a mitochondrial protein of unknown function. It is disrupted more by low molecular weight drugs (*P *< 0.02).

We identified a large number of proteins that we term 'sensitive' to a particular drug property (Table 1). These proteins had different fitness defects after treatment with drugs with either a high or low value of a particular descriptor (Methods; Additional file 6). As an example, YGL084C is involved in glycerol transport. Interestingly, YGL084C is also MlogP-sensitive (*P *< 1^(-4)^) as might be expected for a protein whose main function is the transport of a highly hydrophobic molecule (Figure 5c). Similarly, YAL010C is responsible for the assembly and import of beta barrel proteins and was shown to be aromatic-ring sensitive (*P *< 0.01) (Figure 5b). Finally, YAL008W is a mitochondrial protein of unknown function that showed a preference for smaller drugs (*P *< 0.02) (Figure 5a).

**Table 1:** Number of proteins sensitive to each small molecule descriptor

	MW	Ms	nAB	ARR	Hy	MlogP
MW	77	170	119	143	153	261
Ms	9	102	136	162	158	253
nAB	5	13	47	95	126	229
ARR	4	10	22	70	145	253
Hy	6	26	3	7	82	249
MlogP	12	45	14	13	29	196

Matrix showing the total number of proteins sensitive to each drug property. For each drug property pair (row, column), we report both the number of proteins that are sensitive to both properties (lower triangle, intersection) and the total number of proteins sensitive to either property (upper triangle, union). The diagonal is the total number of proteins that were sensitive to the particular drug property.

We identified numerous other cross patterns that we discuss in more detail below. They are summarized in Figure 5 and Table 1.

### Direct properties of small molecules are sometimes mirrored by those of their protein targets

In order to disrupt a protein's function, a small molecule must either bind directly to the protein or act indirectly by interfering with another component up or downstream. In the former case, there is a logical intuition that the composition of the small molecule would constrain the types of proteins that it could affect or that certain properties of a small molecule would be more favorable in disrupting a particular type of target proteins. Using the GRAVY score (a standard means of measuring protein hydrophobicity) [25], we found that the 102 charge-sensitive proteins were more hydrophobic in nature (Welch's t-test *P *< 0.05) than the charge-insensitive proteins. Since low charge compounds would be expected to more easily interact and thus more easily disrupt the function of membrane proteins, this finding is concordant with membrane protein physiology.

In addition, the seventy AR-sensitive proteins had a higher degree of aromaticity than the AR-insensitive set (*P *< 0.05). Such compounds would be particularly effective in disrupting aromatic proteins because of their ability to disrupt stacking interactions.

### Localization constrains physicochemical properties of drugs

Since a small molecule must be able to reach its protein to disrupt function, the localization of the protein will have a profound effect restricting the entrance of compounds with one set of physicochemical characteristics and enhancing favorable access of others. Likewise, topological properties of the networks, such as degree, can be used to infer additional constraints on the physicochemical property of the drugs [26]. Using CRIT, we identified global cross patterns between the physiological conditions encountered in the protein's compartment and the compound's corresponding physicochemical properties. Proteins that responded differently to drugs that were charged as opposed to those that were uncharged, are more likely to localize to the Golgi (highly hydrophobic) or the nucleus than proteins which were as affected or unaffected by charged as with uncharged drugs (charge-insensitive proteins).

We identified forty-seven proteins that were sensitive to compounds containing aromatic bonds (AB-sensitive proteins) and showed that these proteins have a tendency to be localized to mitochondria and vacuoles. From this cross pattern, one could infer that access to mitochondrial or vacuolar proteins is partially determined by the aromatic nature of the compound. Interestingly, a recent drug screen identified six highly aromatic compounds as being particularly effective in modulating these mitochondrial functions [27].

Further, we found that AR-sensitive proteins had higher degree in the regulatory interaction network reinforcing the importance of disrupting aromatic interactions in this class of proteins.

### GO-specific disruption

To understand what features underly disruption of a particular functional class (for example cell wall synthesis), we calculated the GO enrichment [28]. We found enrichment in RNA metabolism for both AR and AB-sensitive proteins and in DNA binding for AR and hydrophilicity-sensitive proteins. In addition, charge-sensitive proteins showed an enrichment in transferase activity and MlogP in transcriptional regulator activity and protein catabolism. Thus, suggesting a specific functional class can be related to the compounds' physicochemical properties.

### Environmental stress response

In a study by Gasch *et al.*, it was shown that there is both a 'core' of yeast genes that respond in a characteristic manner to a diverse array of stresses and a set that respond in a stress-specific manner [24]. We applied CRIT to investigate whether molecular properties can reveal similarities that unify common stress responses or conversely provide a more mechanistic reasoning for the observed specificities (dissimilarities) in responding to stress.

We observed structural feature-specificity in a number of yeast genes including TOR1, CYC7, GPM2, and SSA3 with known stress-specific responses (Additional file 7). As an example, TOR1 (protein of rapamycin) is a kinase that controls response to amino acid starvation, and it also exhibits a sensitivity to a compound's charge (*P *< 0.04). Similarly, SSA3, involved in protein unfolding and heat shock response, is MlogP-sensitive (*P *< 0.01). One intriguing possibility is that one can use the connection with specific drug features to track an underlying molecular reasoning for similarities and conversely dissimilarities in stress response.

One of the hallmarks of the general environmental stress response (ESR) in yeast is that only one of a pair of isozymes may have a role in stress response at all, or both may have roles but each under a different set of stress conditions [29]. It is possible that isozymes' subtly different amino acid sequences results in dissimilar biochemical properties that may render one isozyme more suitable than another under a given set of conditions. We observed differential drug property sensitivities between several pairs of isozymes (Additional file 7). The non-ESR regulated glutathione transferase, GTT1, exhibits charge sensitivity (*P *< 0.01), but GTT2 showed no specificity in its response to drug treatments. This suggests that differential drug sensitivity may prove useful in tracking these underlying biochemical differences and how they impact stress response regulation.

Finally, it has been shown that different perturbations can sometimes induce the same type of stress [30]. As an example, oxidative stress can be triggered in yeast through the application of either hydrogen peroxide or menadione among others [31]. We identified a cross pattern between MlogP and hydrogen peroxide treatment; however, we found no significant cross pattern between the MlogP and the menadione profile. Interestingly, differential response to hydrogen peroxide, menadione, and two other types of oxidants was observed in *S. pombe *[32]. Differences in structural parameter sensitivities may reflect the specific requirements in responding to each of the different types of reactive species generated. Thus, cross patterns may prove useful in teasing apart differences between closely related stress responses.

### Guilt by association to predict function or mechanism of compound action

CRIT is able to generate testable hypotheses related to predicting function and mechanism of compound action. Akin to building a compendium of a protein's response to small molecules, the cross patterns described can also be aggregated to generate a profile of a protein's sensitivity to drug properties across a number of different small molecule applications (drug property-sensitivity profiles). Including additional features of these small molecules can allow sophisticated structure-based profiles to be built (Additional file 5, 6) allowing for possible inference of function. Using just these six well-characterized molecular descriptors, we see evidence that proteins whose sensitivity profiles overlapped were also functionally similar. Thus, it is likely that by applying traditional guilt-by-association rules using these profiles [33], we can generate hypotheses about the role of uncharacterized proteins, such as YCR101C, which is both molecular weight (*P *< 0.05) and aromatic-bond sensitive (*P *< 0.03). Five proteins had a similar DP-sensitivity profile to YCR101C including the glycerol transporter YGL084C. The shared DP-sensitivities also mapped to osmotic stress response and a proclivity to be localized to the vacuoles. The physiological role of the vacuole during osmotic stress is unclear; however, it is known that phosphoinositides quickly accumulate stimulating actin patch-formation and that disruption of this pathway causes abnormal vacuole morphology. Based on these observations, we would suggest that YCR101C plays a role in cytoskeletal reorganization in the vacuole.

### Generality of CRIT

The amount of available multidimensional data will continue to grow. A number of current datasets can be formulated in terms of connector matrices and thus be amenable to the CRIT framework. The derivation of the connector matrix can be trivial such as mapping transcription factors to their binding sites or splice sites to their corresponding gene. However, the real power lies in more subtle mappings. As an example, metagenomics provides a catalogue of nucleotide sequences for an environment. Genes derived from these datasets have not only a specific function but also environmental context. Thus, using such a connector matrix provides the potential to identify more subtle connections between properties of genes and analogously, properties of the sites the genes are derived from (for example temperature). Similarly, whereas direct integration only allows for identification of tissue-specific or tumor-specific expression, CRIT can connect more global properties of tissues to sets of gene properties or metabolites as it preserves the direct connection between features. CRIT in theory is not limited to three levels. As an example, one can integrate clinical state alongside a person's microbial community structure. Such responses can then be linked to specific metabolites, and the interaction between the human and microbial metabolite complements and its effect on disease progression could be mapped. However, currently available datasets are not yet amenable to this treatment. Further, one caveat of such cascades is that although the means to evaluate the significance of each individual step of CRIT is well understood, generation and evaluation of such complex chains of inferences requires further investigation. We have begun such an investigation through the use of synthetic datasets, but only further experimental and computational characterization can reveal the true utility and justification for integration in such high dimensional space. Further, we have discussed only the simplest implementation of CRIT as a framework for the exploration of such multidimensional data integration.

## Conclusions

At the moment, yeast represents a special case in terms of the range of available system-wide datasets; however, yeast is a harbinger for other systems. Technological and computational advances are leading to a dramatic increase in system-wide datasets for many model organisms. The unprecedented scale and diversity of these datasets present both opportunities for new discoveries and interesting computational challenges. Straightforward integration, as currently done in genomics, does not provide enough flexibility when the dataset can no longer be indexed on a gene or protein or even a single class of variable. We have introduced a method to discover cross patterns between differently indexed metadata. We applied CRIT to identify cross patterns connecting small molecule descriptor sensitivities to disparate types of systems-wide and transcription factor features to features of those their target genes. Further, we showed that this type of integration can reveal novel and non-obvious connections between many different and not necessarily gene-centric types of data. In a broader context, to fully leverage the coming deluge of systems-wide datasets will require the development of new types of spanning techniques as more model organisms join the ranks of yeast in terms of both quantity and diversity of data. Mining such complexity requires a robust infrastructure and new computational models.

## Materials and methods

### Formal definition of CRIT

CRIT requires at least three matrices *M*^1^, *M*^2^, and *M*^3^, although conceptually it can be applied to *n *matrices. We indicate the set of rows and columns indexing a matrix by using capital letters, for example *M*[*I*, *J*] is a matrix whose rows and columns are indexed by the sets *I *and *J*, respectively. *M*[*i*, *j*] is the element at row *i *and column *j*.

It is required that the columns of each matrix are indexed over the same set as the rows of the next. Thus, we refer to the nth matrix's rows as *I*^*n*-1 ^and its columns as *I^n^*, instead of *I *and *J *as above. The (*n *+ 1)th matrix's rows would then be *I^n^*, giving the desired correspondence between the columns and rows of adjacent matrices. The sequence of matrices our algorithm operates on is thus:

We label the columns of each matrix, and refer to these as *L*^1^, *L*^2^, ..., *L^n^*. As an example, consider.(1)

so that *L*[1] = *a*, *L*[2] = *b*, and so on. Given such a vector it will be necessary to extract the set of indices that are assigned the labels *a *and *b*. If the vector is indexed by *I *= {1,2,3,4}, then we would have  and . The hat notation *Î *just reminds us that these sets are subsets of *I*.

Given a labeling *L*^*n*-1 ^for matrix *M*^*n*-1^, we can then immediately transfer the labels to the rows of matrix *M^n ^*since they are indexed by the same set.

The next step is to slice *M^n ^*along its rows such that each resulting partition has only rows with one label.

For example,  gives the indices labeled *a *by the previous matrix and so the slice  gives just those rows of the current matrix that were labeled *a *by the previous.

Finally, let *f^n ^*denote the discriminator function employed to label the columns of *M^n^*. It first partitions the rows of *M^n ^*by each label obtained from the previous matrix. It then considers whether each column *j *of *M^n ^*differs amongst these row slices, and sets *L^n^*[*j*] to *a *or *b *accordingly. The method for determining whether columns 'differ' is dependent on the specific problem, and we will discuss the standard statistical techniques we used in the particular applications investigated in this work. Our framework is meant to be general and does not restrict the choice of statistical methods that can be employed. Tests that partition the columns into more than two sets could also be employed.

In essence, the *n*th discriminator takes as input matrix *M^n ^*and the previous labeling *L*^*n*-1^, and it returns a new labeling *L^n^*. In other words, *L^n ^*= *f^n^*(*M^n^*, *L*^*n*-1^), for *n *= 1, 2, ..., *n*.

The final output of the algorithm defines a new type of relationship between a row *i *∈ *I*^0 ^of the initial matrix and a column *j *∈ *I^n ^*of the final matrix such that *j *is labeled as being *interesting *(according to the particular application) through the propagation of labelings from *L*^0 ^through *L^n^*. We call such relationships cross patterns. We notate the set of cross patterns between all the rows of the initial matrix and all the columns of the final matrix by *I*^0 ^↦*I^n^*. The specific cross pattern would be defined as *i *↦ *j*.

On the first iteration, numbered 1, an initial labeling *L*^0 ^must be obtained from an external procedure. In the next section, we show that our specific application of CRIT does not require this, or alternatively that it consists trivially of a single label. Thus, the initial discriminator *f*^1 ^differs slightly in that it does not compare values between multiple slices, but uses another test to assign labels to the first set of columns. CRIT considers each feature separately. Thus if two features are correlated they will each generate a cross pattern, and both will be agged as significant. The decision of how to treat such features is left to the user.

### Pseudocode

M_1, M_2, ..., M_n = load matrices from data file

L_0 = compute initial labeling using custom method

for i in 1.. n do

Ihat_a_(i-1) = indices labeled 'a' in L_(i-1)

Ihat_b_(i-i) = indices labeled 'b' in L_(i-1)

L_i = ttest(M_i[Ihat_a_(i-1), J], M_i[Ihat_b_(i-1), J])

done

Above we have written simply ttest but a different statistical test can be used on each iteration of the loop and in fact the tests should be selected as appropriate for the specific data being studied. Also we have used the labels 'a' and 'b' but more intuitive names are used in the main text. When the loop is complete, we have the final labeling L_n. Depending on the particular propagation of labels that is relevant for the specific application, we can now see which of the initial rows of M_1 are related to the columns of M_n.

## Abbreviations

CAI: codon adaptation index; CCA: canonical correlation analysis; CRIT: cross pattern identification technique; DP: drug property; ESR: environmental stress response; ER: estrogen receptor; FOP: frequency of optimal codons; PCA: principal component analysis; TF: transcription factor.

## Authors' contributions

TG designed the experiments and completed the analysis. AA and TG developed the formalism. TG wrote the bulk of the manuscript with contributions from AA, MS, and MG. MS and MG provided overall guidance. All authors read and approved the final manuscript.

## Supplementary Material

Additional file 1 **Supplementary materials**. Further description of methods in the text and results of synthetic cross patterns. Click here for file

Additional file 2 **Table of TF-target properties**. Full listing of transcription factor and gene target properties from the regulatory network example. Click here for file

Additional file 3 **Table of SMILES**. 291 small molecules and their SMILE representations. Click here for file

Additional file 4 **Table of molecular descriptors index**. Index of all molecular descriptors calculated (only six used in main text). Click here for file

Additional file 5 **Table of molecular descriptors values**. The values for all the molecular descriptors calculated (only six used in the main text). Click here for file

Additional file 6 **Table of sensitivity scores for each drug-protein treatment**. Listing of the sensitivity score for each protein for each of the six molecular descriptors used in the text. Click here for file

Additional file 7 **Summary table of the findings from the environmental stress response**. Click here for file
