# Supplementary material for: The CRIT framework for identifying cross patterns in systems biology and application to chemogenomics
Source: Genome Biol. 2011 Mar 31;12(3):R32. doi: 10.1186/gb-2011-12-3-r32 (PMC3129682; doi:10.1186/gb-2011-12-3-r32)
Supplement: Additional file 1 — Supplementary materials. Further description of methods in the text and results of synthetic cross patterns. [file gb-2011-12-3-r32-S1.PDF]

Supplementary Materials

Gianoulis, et al

Table Legends

Tables provided as separate files.

Supplementary Table 1 Full listing of transcription factor and gene target properties from the regulatory network example.
Supplementary Table 2 The 291 small molecules and their SMILE representation.
Supplementary Table 3 The index of all molecular descriptors calculated (only 6 used in the main text).
Supplementary Table 4 Values for all molecular descriptors.
Supplementary Table 5 Listing of the sensitivity score for each protein for each of the 6 molecular descriptors used in the text.
Supplementary Table 6 Summary table of the findings from the environmental stress response.

Synthetic Dataset Generation and Results

The goal of the synthetic datasets was two fold (1) identify the point at which noise would obscure a perfectly hard wired cross pattern and (2) conversely, identify the point at which perfectly split data would become identified as a cross pattern. In essence, the cross pattern is controlled by the connector matrix. By altering the connections, we can explore the effect of noise on the system.

This is best illustrated with an example as shown in Supplementary Figure 1. In this case, we have hard wired a cross pattern between the charge of a drug and the charge of a protein by doing the following. In every case where the protein is charged and the drug the protein is treated with is charged, we entered a 1 in the connector matrix to indicate an interaction between the protein and the drug. Conversely, in all instances where the drug is not charged, we entered a 0 indicating that there is no interaction between the protein and the drug. Next, we randomly flipped increasing numbers of bits to simulate the addition of noise. After each iteration, we computed a p value using the Fisher Exact Test, and plotted the p value vs the noise level Supplementary Figure 1.

We then constructed a second dataset where there was no relationship between being protein and drug charge by splitting the number of interactions equally between charged and uncharged drug treatments. As in the first dataset, we then added noise to the system by flipping bits.

The plot in Supplementary Figure 1B shows the results of these two simulations. The hard wired cross pattern seems quite robust to noise. Only after fifty percent of the data was corrupted do we start to observe significant degradation. In addition, it is quite hard to turn a non-cross pattern into a cross pattern. Forty percent of the data needed to be corrupted before we could recover a cross pattern from the perfectly split data.

Randomization Results

We shuffled the labels for each drug treatment. Next, we partitioned the drug-protein matrix into two slices on the basis of this randomized label. For each of the 1200 proteins, we assessed whether the random label was discriminatory ($p < .05$). We found after 100 trials only two proteins were considered "sensitive" to their random label.

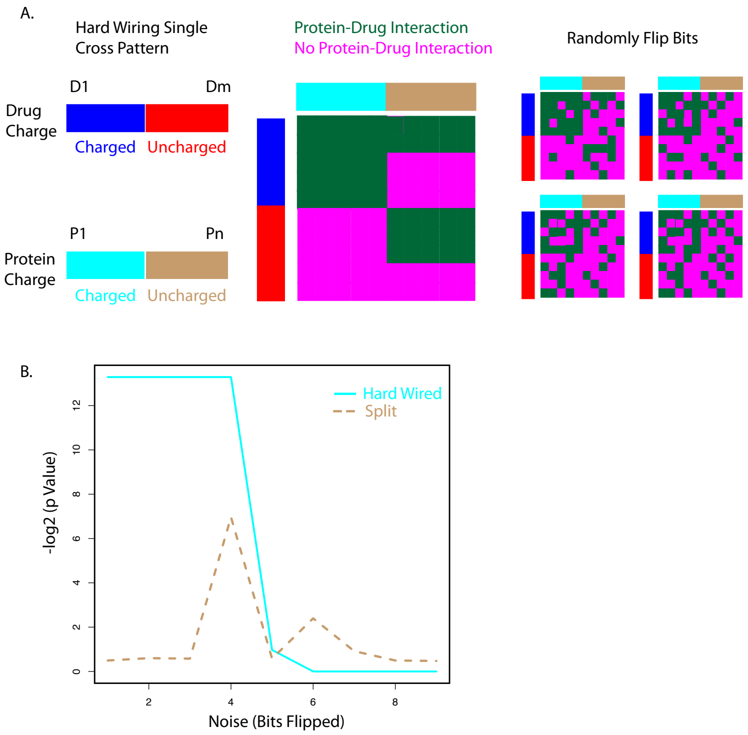

Figure 1: Results of synthetic dataset.

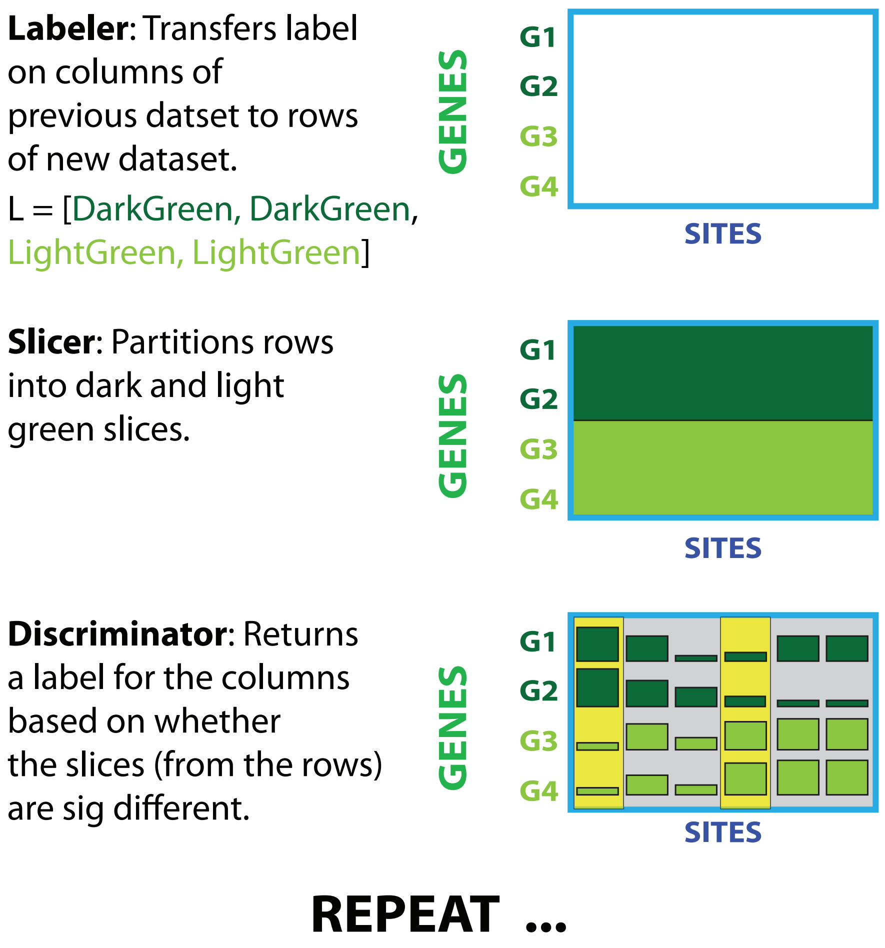

Figure 2: Schematic of CRIT's labeler, slicer, and discriminator.

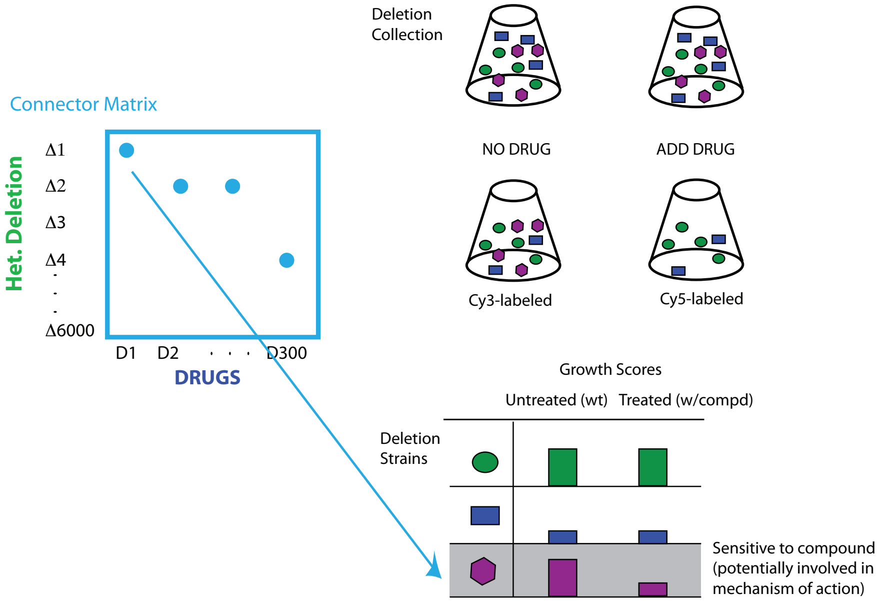

Figure 3: Cartoon describing fitness profiling experiment. See Hillenmeyer Science 2008 for more technical details.

Pseudocode

```
M_1, M_2,..., M_n = load matrices from data file
L_0 = compute initial labeling using custom method

for i in 1..n do
  Ihat_a_(i-1) = indices labeled ‘a’ in L_(i-1)
  Ihat_b_(i-i) = indices labeled ‘b’ in L_(i-1)
  L_i = ttest(M_i[Ihat_a_(i-1), J], M_i[Ihat_b_(i-1), J])
done
```

Above we have written simply `ttest` but a different statistical test can be used on each iteration of the loop and in fact the tests should be selected as appropriate for the specific data being studied.

Also we have used the labels ‘a’ and ‘b’ but more intuitive names are used in the main text.

When the loop is complete, we have the final labeling `L_n`. Depending on the particular propagation of labels that is relevant for the specifical application, we can now see which of the initial rows of `M_1` are related to the columns of `M_n`.

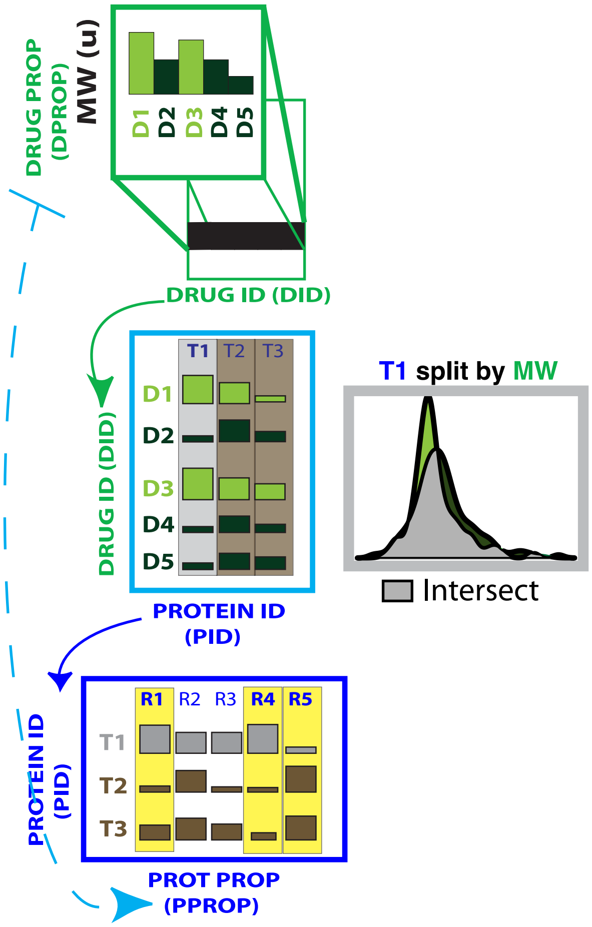
DRUG PROP
(DPROP)
MW (u)
D1
D2
D3
D4
D5
DRUG ID (DID)
T1
T2
T3
D1
D2
D3
D4
D5
DRUG ID (DID)
PROTEIN ID
(PID)
T1 split by MW
Intersect
R1
R2
R3
R4
R5
T1
T2
T3
PROTEIN ID
(PID)
PROT PROP
(PPROP)
