# Supplementary material for: The CRIT framework for identifying cross patterns in systems biology and application to chemogenomics
Source: Genome Biol. 2011 Mar 31;12(3):R32. doi: 10.1186/gb-2011-12-3-r32 (PMC3129682; doi:10.1186/gb-2011-12-3-r32)
Supplement: Additional file 7 — Summary table of the findings from the environmental stress response. [file gb-2011-12-3-r32-S7.PDF]

Gasch Results	Our Results	Implications	Example
**Specific** sets of genes have coordinated behaviors in response to disparate types of stress (ESR-regulated).	ESR-regulated genes exhibit **specific** DP-sensitivities.	DP-sensitivities may allow the tracking of an underlying molecular reasoning for similarities and dissimilarities in ESR. *That is, they may form Equivalence Groups with Env Stress.*	TOR1, CYC7, GPM2, SSA3 stereotypical stress response and DP-sens.
Isozymes are often involved in **different** types of stress response.	Isozymes exhibit **different** DP-sensitivities.	Subtle differences in amino acids *may render one isozyme more suitable than another* under a given set of conditions. DP-sensitivities may allow tracking of underlying biochemical differences.	GGT1 exhibited charge sensitive, but GTT2 showed no specificity
Different treatments resulting in the same type of stress often work through the **same** stress response pathway.	Different treatments resulting in the same type of stress exhibit **different** DP-sensitivities.	Different mechanisms of similar ESR can be detected vi differential DP-sensitivities.	H202 and meniadione have different DP-sensitivities and subtle differences in their response to osmotic stress have since been identified.
